# The role of URO17™ biomarker to enhance diagnosis of urothelial cancer in new hematuria patients—First European Data

**DOI:** 10.1002/bco2.50

**Published:** 2020-10-20

**Authors:** Nikhil Vasdev, Alexander Hampson, Samita Agarwal, Rajiv Swamy, Michael Chilvers, Amy Hampson, Sholeh Jahanfard, Nam Kim

**Affiliations:** ^1^ Hertfordshire and Bedfordshire Urological Cancer Centre Department of Urology Lister Hospital Stevenage UK; ^2^ School of Life and Medical Sciences University of Hertfordshire Hatfield UK; ^3^ Department of Pathology Lister Hospital Stevenage UK; ^4^ Department of Anaesthetics Lister Hospital Stevenage UK; ^5^ KDx Diagnostics Inc Campbell CA USA

**Keywords:** biomarker, bladder cancer, cytology, diagnostic, oncology

## Abstract

**Introduction and objectives:**

Novel biomarker research is vital for the progression of safe and thorough diagnostic medicine. There is now a need to improve the diagnosis of bladder cancer via a noninvasive urine test while balancing the risks of harm from investigational procedures, such as cystoscopy and radiological tests, against the likelihood of malignancy. We evaluate the diagnostic accuracy and sensitivity of Uro17™ urinary biomarker for the detection of urothelial cancer in hematuria patients in a prospective blinded validation study. Uro17™ is an immunobiomarker which binds to the oncoprotein Keratin 17, which is involved in the replication cycle of malignant cells. This study compared cystoscopic and histological investigations against Uro17™ results in patients being investigated for symptoms of urothelial cancer.

**Materials and methods:**

After receiving both local and national ethics/protocol approval, 71 patients were consented and recruited into the study. All patients were scheduled to undergo cystoscopic investigation, and following recruitment, a urine sample was collected. Urine samples were anonymized and processed as per standard cytology protocols and stained using Uro17™ immunobiomarker. The pathologists assessing the results were blinded to the patient and background history, and the results were compared to the biopsy histology.

**Results:**

The full cohort of enrolled patients consisted of 71 participants included. There were 55 males and 16 females, with an average age of 70. Thirteen were current smokers, 42 ex‐smokers, and 16 nonsmokers. The malignancies detected included both muscle‐invasive (n = 6) and non‐muscle‐invasive tumors (n = 38), and tumors of all grades and carcinoma in situ. Uro17™ was shown to have an overall sensitivity of 100% and a specificity of 92.6%, with a positive predictive value of 0.957 and negative predictive value of 1. Uro17™ investigation was positive in every case of urothelial malignancy.

**Conclusions:**

Our current data indicates Uro17™ is a highly sensitive noninvasive bladder cancer urine detection test that can improve the diagnosis of Bladder cancer. This can further improve diagnostic capabilities in primary care, reduce the number of referrals to Urology department, and reduce the number of unnecessary invasive procedures for new patients with a suspected urinary bladder cancer.

## 1 INTRODUCTION AND OBJECTIVES

Annually, there are over 197 000 new diagnosis of bladder cancer in Europe and 430 000 globally, making it the fourth most prevalent malignancy in men and the fifth most prevalent in women.^1^, ^2^ Prognosis and mortality is strongly correlated with cancer staging at the time of diagnosis, with muscle invasion (T2‐4) resulting in a significant increase in mortality.^3^ Unfortunately, 37% of new cases are found to have muscle‐invasive malignancies at the time of diagnosis which can result in a 60% 5 year survival rate, dropping to 4% for metastatic malignancy.^4^ However, new developments in the management of both nonmetastatic and metastatic disease continue with improvement in survival.^4^, ^5^ This emphasizes despite of the new developments is on the importance of early diagnosis and timely treatment, being the clear clinical priority.

The UK currently does not currently have a screening program for bladder cancer. In a majority of patients the diagnosis and investigations for bladder cancer rely on symptomatic presentation to general practice (Visible/Non‐visible hematuria) or incidental findings. The UK National Institute of Health Care and Excellence (NICE) guidance recommends that community physicians refer urgently to the urology department for suspected bladder cancer based upon symptoms of hematuria and age, shown in Table 1.^6^

**TABLE 1:** NICE Referral guidelines for suspected bladder cancer^6^

Age criteria	Symptom criteria
Aged 45 and over	Unexplained visible hematuria without urinary tract infection
Aged 45 and over	Visible hematuria that persists or recurs after successful treatment of urinary tract infection
Aged 60 and over	Unexplained non‐visible hematuria and either dysuria or a raised white cell count on a blood test

While visible hematuria is a significant predictor of bladder cancer, associated with an odds ratio of 34, the detection rate of malignancy in patients who present with visible hematuria has been reported as 18.9%, making this symptom not only nonspecific, but also less sensitive, and differential diagnoses may result in uncertainties or delays in referrals from primary care.^7^ Visible hematuria has been shown to only be present in around half of bladder cancer cases, and other symptoms are nonspecific, such as abdominal pain and constipation, or even potential “red herrings” such as raised inflammatory markers and urinary tract infections.^8^ There is a clear need for improved diagnostic methods, especially for patients with bladder cancer who may present without visible hematuria.^3^

The gold standard for investigation of bladder cancer is cystoscopy, as this allows direct visualization of the urothelial lining of the urethra and bladder, however, this is uncomfortable for the patient and does not allow investigation of the upper tracts.^9^ Cystoscopy is often paired with radiological scanning such as CT urography to assess for upper urinary tract disease. This is usually performed during the first encounter that the patient has with the urology service, resulting in a significant number of patients unnecessarily undergoing both radiation exposure and invasive procedures, which can lead to further complications such as urinary tract infections.^10^, ^11^ These cases of unnecessary investigations for bladder cancer result in significant costs, estimated to be over £100 million annually in the UK alone.^12^

Following treatment for diagnosed bladder cancer, follow‐up by means of cystoscopic surveillance is required for many years. For non‐muscle‐invasive bladder cancer (NMIBC), the 3 year total costs per person for surveillance alone are estimated to be over £4500, with additional costs for any recurrence or progression.^13^

Risk grouping/stratifying models, probability nomograms, and artificial neural networks are all methods by which urologists have hoped to reduce the amount of unnecessary investigations that are performed and to help better focus resources on patients who truly require them.^14^ However, these methods, no matter how much collective data are inputted, are generic and not patient specific, and will therefore result in some patients being incorrectly stratified.

As research into the molecular biology of bladder cancer has progressed, genetic markers have been identified in the tumorigenesis and progression of the disease.^15^ This research has translated into identifiable biomarker that could be used to identify bladder cancer from urine or blood samples. Urine samples lend a significant advantage as they will be in physical contact with any malignancy and are easily obtainable.

A need for an inexpensive diagnostic test or screening tool has been expressed repeatedly.^7^, ^12^, ^16^ In 2008, there were six urine‐based biomarkers FDA cleared/approved for the surveillance of bladder cancers which were reviewed by Herman et al., but these were all found to have sensitivities equal to or only slightly greater than cytology alone.^17^

In 2013, Casadio et al. investigated cell free DNA in urine samples as a potential biomarker.^18^ It was shown to have a high predictive value for early NMIBC with sensitivity of 0.73 and specificity of 0.83, but due to being a specialized investigation and having high processing costs, it was unsuitable to be used as a feasible screening or diagnostic tool.^18^

A biomarker that has been researched extensively is the nuclear matrix protein NMP22, which is involved in mitosis and is often raised in urine samples where malignancy is present. The test is performed using monoclonal antibody testing with ELISA meaning that it could be accessible and affordable on a large scale. A meta‐analysis of 19 trials was performed by Wang et al. which showed sensitivity and specificity were 56% (52%‐59%) and 88% (87%‐89%), respectively.^19^

UroVysion™ is another FDA‐approved urinary test from recent literature. It functions by utilizing fluorescent in situ hybridization (FISH) to identify chromosomal abnormalities characteristic of bladder cancer such as deletion 9p21 and alterations to chromosome 3, 7, and 17.^20^ FISH requires gene‐specific probes to allow detection of these genetic changes, and then, direct visualization with the use of a fluorescent microscope which, while costing less that cystoscopy, is relatively high cost when compared with other biomarkers. It also requires specialist personnel to interpret results.

Keratin 17 (K17) is a member of cytokeratin family of proteins normally expressed in hair follicles and nail beds.^21^ Previous studies have shown that K17 is an oncoprotein associated with poor prognosis in tumorigenesis of many malignancies including cervical, endometrial, esophageal, lung, and bladder.^22^, ^23^, ^24^ K17 has already been investigated for its biomarker possibilities for the detection and prognosis of cervical cancer.^24^

Recently, Babu et al. performed a study looking at K17 in urothelial carcinomas and showed that K17 is expressed in urothelial carcinomas at levels of 2.5 to 8 log fold greater than normal urothelial mucosa, making it a promising biomarker for bladder cancer.^25^ In this study, analysis of biopsy tissue blocks showed that K17 was expressed in all grades and stages of urothelial cancers tested (PUC‐LG, PUC‐HG, and UC).^25^ More importantly, this study also examined the expression of K17 in urine cytology samples from 112 patients undergoing routine bladder cancer monitoring and the study showed that K17 has sensitivity of 100% and specificity of 96% in identifying patients with recurrent urothelial cancers. However, in the study, expression of K17 in newly diagnosed cancers from an hematuria population was not examined.

In the current study, we for the first time report the result of K17 expression in urine cytology samples from hematuria patients with no prior history of urothelial cancer to evaluate application of K17 in identifying urothelial cancer in patients with hematuria.

## 2 PATIENTS AND METHODS

### 2.1 Aims and objectives

The aim of this trial was to investigate and measure the accuracy and precision of the urinary bladder cancer test, URO17™, which uses K17, when compared with the gold standard of rigid cystoscopy and bladder biopsy, in new diagnosis of bladder cancer in hematuria patients without a prior history of bladder cancer.

### 2.2 Study design

**FIGURE 1:**
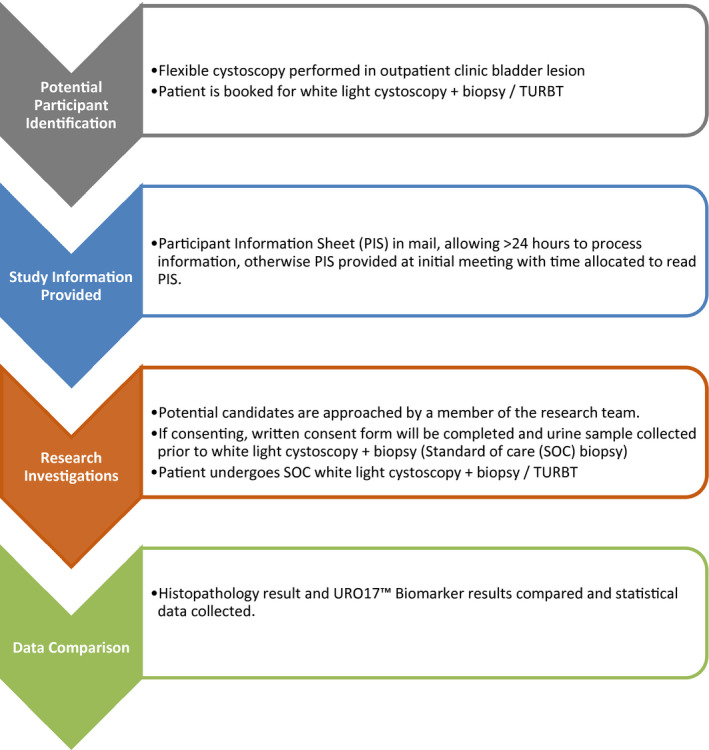
Study design flowchart

The study was designed as a prospective blinded validation trial with 71 participants who were under investigation for new diagnosis of urothelial tract cancer. The study was performed at East and North Herts NHS Trust using input from Urologists, Pathologists, R&D Department, and patient advocates. Study design is demonstrated in Figure 1.

### 2.3 Ethics approval

Local ethics approval gained through R&D departmental procedure. IRAS Application 253585 submitted and national ethics/protocol approval received (18/EE/0395).

### 2.4 Inclusion and exclusion criteria

Inclusion:

- Patients aged ≥18 years of age;
- Patients under investigation for bladder cancer due to undergo investigative standard of care biopsy.

Exclusion:

- Patients aged <18 years of age;
- Patients with catheter in situ;
- Patients who are currently undergoing radiation therapy;
- Proposed subject has no bladder (due to surgical removal);
- Patients unable or unwilling to provide consent;
- Patients currently on investigational drug trials.

As the inclusion criteria required patients to be undergoing cystoscopy & biopsy for histological analysis and comparison, and as our trust utilizes One Stop Clinic Services, the meant that patients had likely already undergone flexible cystoscopy prior to study recruitment. The inclusion criteria were developed this way to ensure that full histological analysis was performed thus reducing possible surgeon variability.

### 2.5 Data collection

Following consenting and recruitment, a urine sample was collected from each participant and the following data were collected; gender; age; weight, height, and BMI; smoking status; previously diagnosis of bladder cancer (staging and treatments provided); urine sample first morning void status; urine sample volume; and urine sample hematuria by routine dipstick. Subsequent cancer diagnosis will be collected for participants for a period of 5 years via review of available patient medical records.

### 2.6 Urine sample preparation

Urine samples were anonymized, stored at 4°C and underwent centrifugation on a Sorvall ST 16 Centrifuge (Thermo Scientific) at 1500 r.p.m. for 5 minutes. The supernatant was discarded, and the pelleted cell deposits were then inspected. If the deposit was hardly visible, a vial of PreservCyt fluid (Hologic) was added into the Universal container, the contents mixed and tipped back into the PreservCyt vial. If the deposit was large but not excessively bloodstained, then, the deposit was mixed using a Pasteur pipette, three drops were added to the PreservCyt vial. If the deposit was excessively blood stained, then, 15 mL of CytoLyt fluid (Hologic) was added to the deposit to wash the sample (this lyses the blood) and the sample underwent centrifugation for 10 minutes at 1500 rpm and three drops of the deposit was added to a PreservCyt vial.

Samples were processed on an automated ThinPrep 2000 processor (Hologic) using non‐gynecological blue filters. This processor generates a ThinPrep slide with a monolayer of cells. Slides were then fixed with Cytofix spray (Hologic) and allowed to dry for 15 minutes.

Sample slides were stored between 2‐8°C and stained in weekly batches using URO17™ (Lot: A8034124, 3.42 mg/mL, 1:5000 dilution in 10% CS) on a Leica detection kit DS9800 for 20 minutes.

### 2.7 Diagnostic test evaluation

The URO17™ staining results were analyzed by two blinded pathologists (SA and RS) simultaneously and results scored as per scoring system in Table 2. No patient information, including any previously diagnosed malignancy of treatments, was available to the pathology team.

**TABLE 2:** URO17™ pathology scoring system

Score		
0—Negative	No stained cells present	
1—Negative	Weak staining (1+)	
2—Positive	Strong staining (2+ and above)	>20 positive staining of cells based on staining intensity

Biopsy histology was performed through the SOC route with no evidence on pathology request systems that patients were part of the trial and were reported according to TNM staging and grading as per WHO classification. This histological data were collected by the research team once URO17™ results had been provided.

## 3 RESULTS

### 3.1 Patient demographics

In the patient cohort (71 included participants), there were 55 males and 16 females, with an average age of 70. 13 (18.31%) were current smokers, 42 (59.15%) ex‐smokers, and 16 (22.54%) nonsmokers, shown in Table 3. The malignancies detected included six muscle‐invasive and 38 non‐muscle‐invasive tumors, and tumors of all grades (9 G1, 14 G2, 18 and G3) and four cases of carcinoma in situ, see Table 4.

**TABLE 3:** Patient demographics

	Patient cohort (n = 71)
Average age (years)	70
Male	55 (77.46%)
Female	16 (22.54%)
Average BMI	27.29
Smokers	13 (18.31%)
Ex‐smokers	42 (59.15%)
Nonsmokers	16 (22.54%)
Visible hematuria samples	11 (15.49%)
Mean sample volume (mL)	20

**TABLE 4:** Malignancies detected

	Patients with malignancy (n = 44)
Ta‐T1 TCC	38
T2‐T4 TCC	6
G1	9
G2	14
G3	18
CIS	4

### 3.2 URO17 results

URO17™ and white light cystoscopy + biopsy results are shown in Table 5. In patients with no previously diagnosed bladder cancer (n = 71), URO17™ was shown to have a sensitivity of 100% and had a specificity of 92.6%. There was a positive predictive value (PPV) of 0.957 and a negative predictive value (NPV) of 1. URO17™ investigation was positive in every case of urothelial malignancy no matter the grade or stage. Smoking status, hematuria, and sample volume had no impact on staining efficacy.

**TABLE 5:** URO17™ and cystoscopy + biopsy results

(n = 71)	Cystoscopy and biopsy (+)	Cystoscopy and biopsy (−)
URO17™ (+)	44	2
URO17™ (−)	0	25

As per the pathology scoring system, Table 2, weak staining cases were classified as negative results. These were specifically reviewed to ensure the scoring system was suitable. Twelve cases were scored with a weak staining, all 12 were negative biopsy results. No interobserver variability was identified between the two pathologist reporting the URO17™ results.

CT Urogram (Triple phase) imaging was performed for every patient as per SOC, 36 had radiological findings clinically suggestive of malignancy of which 28 (77.8%) had positive histology from bladder biopsy. URO17™ was positive in all of the 28 confirmed malignancy cases and was clinically correct in 35 of the 36 radiologically positive cases (97.2%).

## 4 DISCUSSION

This study confirms that URO17™ detected every case of malignancy, no matter the stage, or type of cancer present. A negative predictive value of 1 raises the potential for a significantly effective screening program/referral tool, knowing that URO17™ negative results can be heavily relied upon can significantly reduce the number of unnecessary invasive procedures performed. Our results support previous laboratory studies of K17 which reported sensitivity of 100% and specificity of 96%.^25^

This cohort had a significantly high proportion of malignancy compared to the expected detection rate in visible hematuria cases. As this study required rigid cystoscopy and biopsy for histological analysis, the cohort of patients must have been seen by an urologist in one stop clinic, and most of which would have undergone a flexible cystoscopy. We recognize that this will have resulted in hematuria patients not being suitable for the study as malignancy may have been excluded by flexible cystoscopy but as URO17™ was a novel biomarker, there was an ethical desire to avoid patients undergoing procedures outside of their standard of care. The reassuring data from this trial will influence the design of future larger scale studies and hopefully Ethic Committees will have less issue with patients undergoing cystoscopy based upon URO17™ results.

The patient cohort showed significant level of visible hematuria in their specimen samples, but as this cohort was derived from new patients under investigation, and as the laboratory protocol uses CytoLyt fluid in slide preparation to counteract this, this should not impact the URO17™ test results.

A significant amount of recent biomarker research has been focused on aiding in the surveillance follow‐up of known bladder cancers.^26^, ^27^, ^28^ Unfortunately, as biomarkers are often highly sensitive and can provide a positive result before it is cystoscopically visible, “anticipatory positive results,” the cost effectiveness of combined biomarker‐cystoscopy surveillance, have been shown to be worse than cystoscopy alone.^26^ Focusing on screening adjunct applications for biomarkers with high sensitivity and NPV, such as URO17™, is more likely to result in them becoming cost efficient and valuable adjuncts to both primary and secondary care.

It is important to highlight the significance of these results in comparison to other urinary biomarkers for bladder cancer.^5^, ^19^, ^29^, ^30^, ^31^, ^32^, ^33^ We have confirmed that URO17™ has one of the highest sensitivity and specificity reported in identifying bladder cancer through noninvasive urine samples. Unlike other nucleic‐based bladder cancer biomarker tests that are complex and expensive to run, URO17™ test is an immunocytochemical assay that utilize the same cytology samples that are used in urine cytology. The test is also easily adaptable to the standard IHC instrumentations and reagents that are readily available and familiar in most laboratories which will make the test very a cost‐effective perform. Furthermore, having a same pathologist who usually interpret urine cytology samples also interpret URO17 test will provide efficient workflow and increased utility for urine cytology. While many will see URO17™ as yet another biomarker in a myriad of new noninvasive investigations for bladder cancer, this study confirms the original finding by Babu et al^25^ and expands the application of URO17™ to hematuria patients. In the end, combinations of biomarkers may be the key to providing the most effective investigation where the application of URO17™ will play a major role, and continued research into this must be encouraged with the aim of improving patient diagnostics. We will be following all patients in the study up for 5 years to evaluate those patients who may develop a urological malignancy in due course with a current URO17™ result.

This current data suggest that URO17™ could be a sensitive and specific test to detect PUNLMP and both papillary and nonpapillary carcinomas which could potentially providing diagnostic utility in cases where it could help identify lesions that can be easily missed by traditional urine cytology. Furthermore, the data also showed that URO17™ test was able to detect BC in renal pelvis that was missed by urine cytology and cystoscopy which suggest that URO17™ test could be used to augment and increase the accuracy of cystoscopy and traditional urine cytology in monitoring patients for recurrence. ^5,25^.^5^

This trial was developed with the aim of testing the applicability of URO17™ as a real‐world biomarker for urinary tract malignancy, and therefore, must include all types of patients and malignancy who undergo urological investigations. We constructed this prospective trial in such a way as to allow predictable clinical variabilities, such as urine sample size and sample hematuria, as well as to eliminate confounding factors, such as the skill of a pathologist who may detect non‐biomarker‐related signs in specimens if aware of the background of the patient. As the test uses standard immunostaining available to most pathology departments, we demonstrated that this test could be used by any department/laboratory with minimal difficulties or additional setup.

The next stage of investigation for URO17™ to be rolled out as a screening program would be a large scale, multisite thorough testing to demonstrate these promising results on a national scale.^34^

## 5 CONCLUSION

This study indicates that URO17™ is a highly sensitive (100% in this study) minimally invasive bladder cancer urine biomarker detection test that could help screen patients for bladder cancer in the community with minimal resources required. This can improve worldwide diagnostic capabilities in primary care, efficiently triage patient referrals to Urology department, and could reduce the number of procedures that patients under investigation must go through.

URO17™ urine immunostaining can accurately exclude urothelial malignancy with minimal requirements and will indicate which patients that should be thoroughly investigated. While it would not replace cystoscopy as the gold standard, and histological analysis is still required for staging, URO17™ is an effective adjunct that can help reduced the number of unnecessary cystoscopies in hematuria patients and screen high‐risk patients in the community.
